# Meta-analysis of resting-state fMRI in cervical spondylosis patients using AES-SDM

**DOI:** 10.3389/fneur.2024.1439939

**Published:** 2024-09-24

**Authors:** Qin Zhang, Hui Ding

**Affiliations:** ^1^Guizhou Second People’s Hospital, Guiyang, Guizhou, China; ^2^Guizhou Provincial People’s Hospital, Guiyang, Guizhou Province, China

**Keywords:** cervical spondylosis, neuroimaging, rs-fMRI, AES-SDM, meta-analysis

## Abstract

**Background:**

Resting-state functional magnetic resonance imaging (rs-fMRI) reveals diverse neural activity patterns in cervical spondylosis (CS) patients. However, the reported results are inconsistent. Therefore, our objective was to conduct a meta-analysis to synthesize the findings from existing rs-fMRI studies and identify consistent patterns of neural brain activity alterations in patients with CS.

**Materials and methods:**

A systematic search was conducted across PubMed, Web of Knowledge, Embase, Google Scholar, and CNKI for rs-fMRI studies that compared CS patients with healthy controls (HCs), up to January 28, 2024. Significant cluster coordinates were extracted for comprehensive analysis.

**Results:**

We included 16 studies involving 554 CS patients and 488 HCs. CS patients demonstrated decreased brain function in the right superior temporal gyrus and left postcentral gyrus, and increased function in the left superior frontal gyrus. Jackknife sensitivity analysis validated the robustness of these findings, and Egger’s test confirmed the absence of significant publication bias (*p* > 0.05). Meta-regression showed no significant impact of age or disease duration differences on the results.

**Conclusion:**

This meta-analysis confirms consistent alterations in specific brain regions in CS patients, highlighting the potential of rs-fMRI to refine diagnostic and therapeutic strategies.

**Systematic review registration:**

https://www.crd.york.ac.uk/prospero/, identifier CRD42024496263.

## 1 Background

Cervical Spondylosis (CS) is a prevalent age-related chronic condition characterized primarily by stiffness and pain in the neck and upper back. Due to its high incidence rate, unsatisfactory treatment options, significant healthcare burdens, and impact on quality of life, CS has emerged as a critical public health and societal issue (1). In recent years, the prevalence of CS has been on the rise, becoming a leading cause of non-traumatic spinal cord dysfunction (2). Timely diagnosis and surgical intervention are crucial for alleviating neurological symptoms in CS patients (3). Research shows that chronic pain can lead to the progressive accumulation of brain damage, which may manifest as psychological disorders including depression, anxiety, and sleep disturbances that impact emotional processing. Patients with chronic musculoskeletal pain often experience heightened anxiety, depression, fatigue, and insomnia, which intensify with the severity and multiplicity of pain sites (4). Furthermore, the co-occurrence of anxiety and depression in these patients is linked to significant psychological impairments and reduced quality of life, emphasizing the need to focus on psychological aspects in the management and treatment of chronic pain (5).

Resting state functional magnetic resonance imaging (rs-fMRI) is a commonly used technique to study functional changes in the brains of patients. Analytical methods such as Amplitude of low frequency fluctuation (ALFF) and regional homogeneity (ReHo) are frequently employed. ALFF measures the amplitude of low-frequency fluctuations (0.01–0.08 Hz) in the blood oxygen level dependent (BOLD) signal, providing reliable insights into cortical activity (6). Besides, ReHo assesses the similarity of BOLD signal patterns between a given voxel and its 26 neighboring voxels, offering a different perspective on neural activity (7). ALFF, ReHo, and related algorithms like fractional ALFF (fALFF) play a crucial role in studies with high reliability and specificity on cortical activity, significantly contributing to our understanding of brain function through rs-fMRI technology. Previous studies have indicated functional activity changes in the brains of CS patients in regions such as the superior frontal gyrus, middle frontal gyrus, postcentral gyrus, superior temporal gyrus, insula, sensorimotor area, supplementary motor area, cingulate gyrus, occipital lobe, and precuneus (8–23). However, some studies have reported variability in the functional changes of cortical regions in CS patients, with increased (9, 13), decreased (11, 20), or unchanged activation observed in areas like the precuneus and middle cingulate cortex.

Variability in functional representation across studies may be due to differences in sample sizes, neural activity levels, and imaging parameters. This study uses the Anisotropy Effect Size Signed Differential Mapping (AES-SDM) technique to identify consistent brain function changes and employs a meta-analysis to compare brain region activity between CS patients and healthy controls (HCs), enhancing our understanding of CS’s neurological impact.

## 2 Materials and methods

### 2.1 Literature search

This review was registered with PROSPERO (ID: CRD42024496263). A systematic search for relevant studies was conducted in the PubMed, Web of Knowledge, Embase, Google, and CNKI databases up to January 28, 2024. Keywords included (“cervical spondylopathy” OR “cervical spondylosis” OR “spondylosis” OR “cervical spondylotic”) AND (“regional homogeneity” OR “ReHo” OR “amplitude of low-frequency fluctuation” OR “ALFF” OR “fALFF”) AND (“magnetic resonance” OR “MRI” OR “functional MRI” OR “fMRI”). Manual screening of all potentially related results was also conducted.

Studies were considered eligible based on the following criteria: (1) Clear diagnosis of CS via MRI; (2) absence of pain in other body parts; and (3) reporting of whole-brain results in stereotactic space (MNI or Talairach coordinates) for ALFF, fALFF, dALFF, PerAF, ReHo. Exclusion criteria included: (1) Abstracts, case reports, systematic reviews, meta-analyses; (2) Intervention studies; (3) Studies using only region of interest (ROI) or seed voxel-based analyses; (4) Studies not adhering to CS diagnostic criteria or presenting significant data heterogeneity; (5) Studies without reported coordinates. Two physicians with at least attending doctor qualifications independently conducted the literature search and reached consensus on the results. Study selection was conducted in accordance with the PRISMA guidelines (24) (Table 1).

**Table 1:** Characteristics.

Author, year	Sample size		Age (X ± S)		Man/Female		Field strength	Method	Differential brain region	Corrective methods
	Patient	HCs	Patient	HCs	Patient	HCs				
Zhou, F. 2014 (8)	19	19	49.63 ± 7.36	49.46 ± 7.21	11/8	10/9	3.0 T	ALFF	3	Alphasim
Tan, Y.M. 2015 (9)	21	21	47.95 ± 7.00	47.95 ± 7.00	13/8	13/8	3.0 T	ReHo	10	Alphasim
Tan, Y. 2015 (10)	21	21	47.95 ± 7.00	47.95 ± 7.00	12/9	12/9	3.0 T	ReHo	2	GRF
Yu, C.X. 2017 (11)	25	20	47.68 ± 10.99	42.50 ± 11.94	13/12	10/10	3.0 T	ReHo	8	Alphasim
Chen, Z. 2018 (12)	27	11	57.90 ± 9.10	54.80 ± 8.40	15/12	6/5	3.0 T	ReHo& ALFF	2 & 2	FWE
Chen, J. 2018 (13)	104	96	N/A	N/A	N/A	N/A	3.0 T	ReHo	2	FWE
Xu, Y.K. 2018 (14)	25	20	N/A	N/A	N/A	N/A	3.0 T	ReHo	5	Alphasim
Zhang, CL. 2019 (15)	43	41	49.07 ± 6.73	49.07 ± 6.73	27/16	21/15	3.0 T	ALFF	6	GRF
Kuang, C. 2019 (16)	31	31	54.78 ± 8.41	53.52 ± 8.13	16/17	15/18	3.0 T	zReHo &zALFF	1 & 1	FDR
Yue, X. 2020 (17)	28	25	47.04 ± 8.74	43.56 ± 11.96	17/11	13/12	3.0 T	ALFF	11	Alphasim
Ge, Z.C. 2021 (18)	12	18	55.42 ± 10.58	50.05 ± 11.52	5/7	6/8	3.0 T	ALFF	4	Alphasim
Wu, K.F. 2021 (19)	40	25	49.68 ± 7.13	49.68 ± 7.13	21/19	21/19	3.0 T	ALFF	5	GRF
Fan, N.J. 2022 (20)	44	38	51.3 ± 2.80	51.7 ± 3.60	22/22	20/18	3.0 T	dALFF	3	FWE
Bai, L. 2022 (21)	31	31	51.79 ± 10.21	51.52 ± 9.84	15/16	15/16	3.0 T	ALFF	3	FWE
Zhao, R. 2022 (22)	21	11	53.3 ± 9.13	54.80 ± 8.40	10/11	5/6	3.0 T	zALFF	4	FWE
Su, Q. 2023 (23)	62	60	53.3 ± 7.38	53.4 ± 7.47	31/31	30/30	3.0 T	ALFF	4	FWE

No statistically significant differences were observed in age and gender between CS patients and HCs (*p* > 0.05), as presented in Table 1 of included studies. HCs, healthy controls; ReHo, regional honogeneity; ALFF, amplitude of low-frequency fluctuation; fALFF, fractional amplitude of low-frequency fluctuation; MNI, montreal neurological institute.

### 2.2 AES-SDM analysis

The anisotropy effect size signed differential mapping (AES-SDM) software was used to analyze ReHo/zReHo & ALFF/dALFF/zALFF differences between CS patients and HCs (25). Peak coordinates in Talairach space were converted to MNI space.^1^ If results were presented as z-values, they were converted to t-values for use in the analysis.^2^ AES-SDM reconstructed effect size and statistical parameter maps of increased and decreased brain region activation from individual studies. The Monte Carlo random effects model used in AES-SDM integrated these statistical maps with a significance threshold set at: FWHM = 20 mm, uncorrected voxel *p* < 0.005, and cluster extent ≥10 voxels (26). Cluster coordinate reconstruction involved converting peak t-values to Hedges’ g, followed by application of a Gaussian kernel for nonuniform smoothing of adjacent peak coordinate voxels. Volume rendering of cortical clusters with significant differences was performed in MNI standard space using Mango software.

Egger’s regression test, integrated within the AES-SDM software, was employed to evaluate the effect sizes of peak voxels within significant clusters. Heterogeneity among studies was calculated to assess brain regions contributing to heterogeneity. Jackknife sensitivity analysis was performed to evaluate the robustness of the results by repeating the meta-analysis 16 times, excluding one of the 16 studies in each iteration (27). Funnel plots and meta-regression analyses were created using the peak coordinates of significant cortical activation clusters to assess potential publication bias and the impact of study-level covariates on the observed effects.

## 3 Results

### 3.1 Literature search

**Figure 1:**
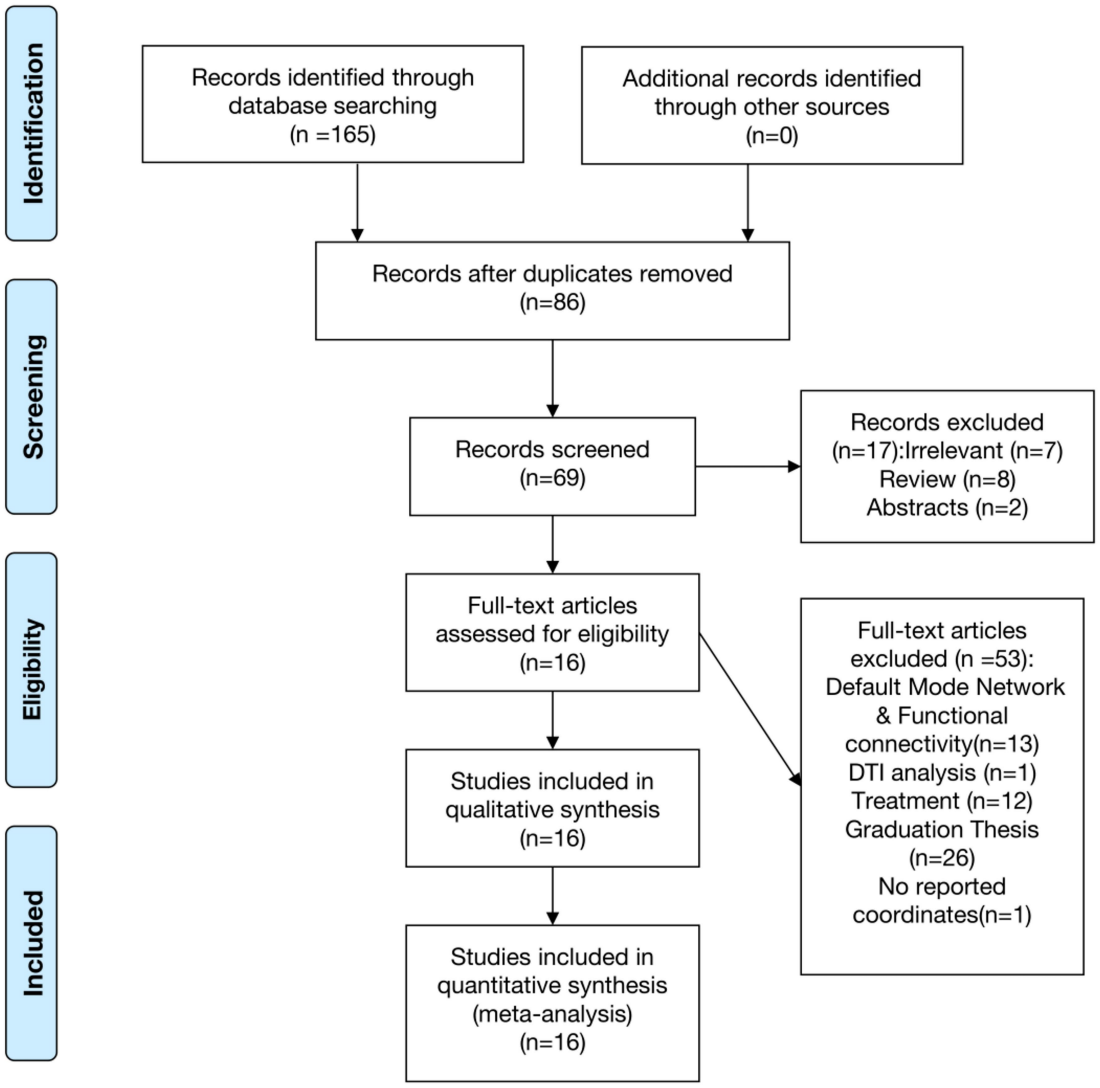
Flow chart of the study selection strategy.

A total of 165 articles were retrieved through the search, among which 16 met the inclusion criteria for the meta-analysis (Figure 1). No additional relevant studies were identified from the references of the selected articles. Various resting-state analysis methods were used across the studies, such as ReHo/zReHo and ALFF/fALFF/dALFF/zALFF. Two papers, Chen (12) and Kuang (16), utilized ReHo and ALFF, zReHo and zALFF methods, respectively, and were considered as separate studies due to the different analytical approaches. Consequently, 16 articles comprising 18 studies were included in the meta-analysis (8–23), involving a total of 554 CS patients and 488 HCs. No statistically significant differences were observed in age and gender between CS patients and HCs (*p* > 0.05; Table 1).

### 3.2 Meta-analysis results

**Figure 2:**
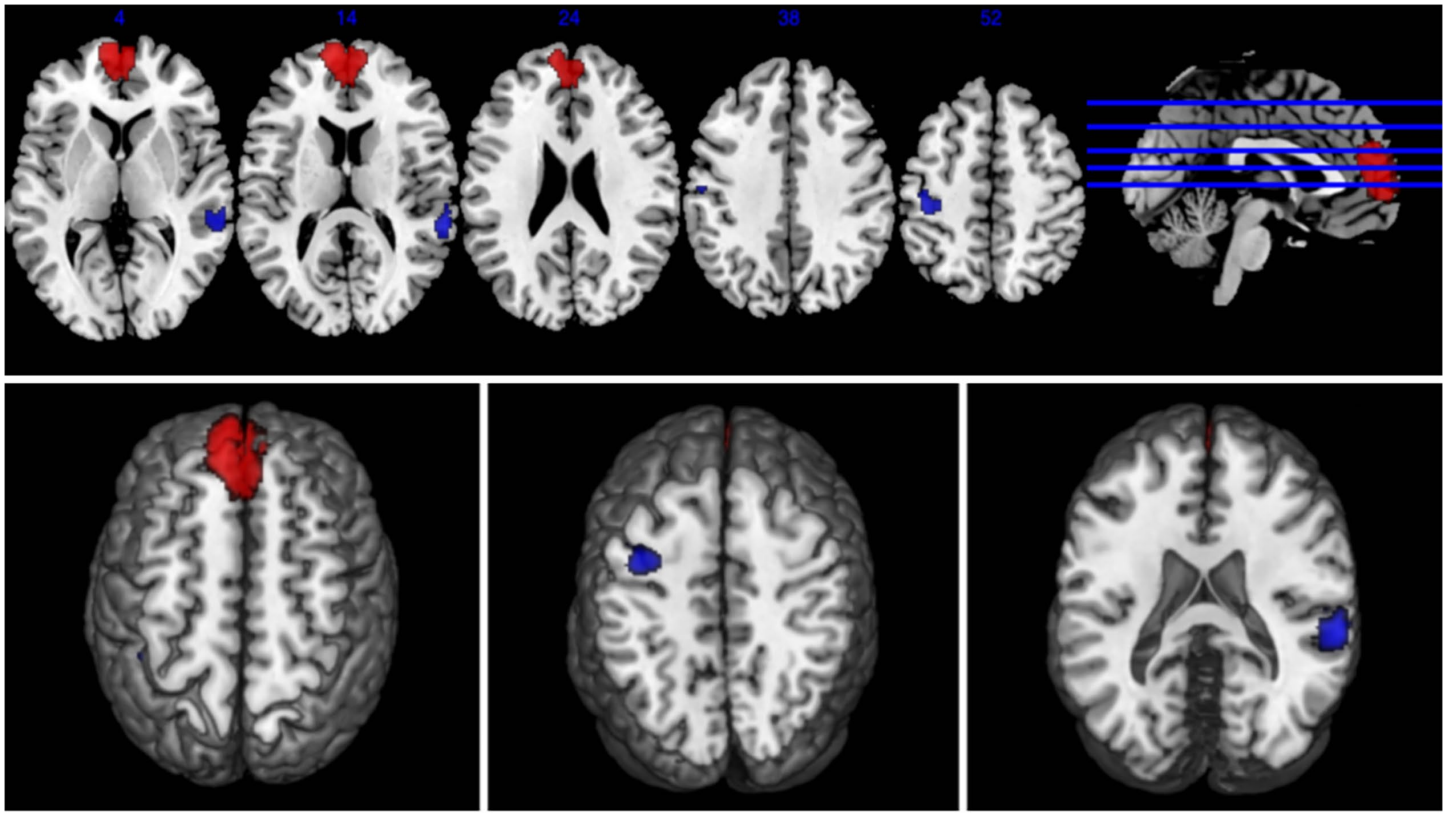
Abnormal regions identified in an SDM-Meta analysis of neuroimaging studies in cervical spondylosis (CS). Regions showing increased activation in CS patients compared to healthy controls (HCs) are highlighted in red, while regions with decreased activation are highlighted in blue.

The results of the AES-SDM analysis are summarized in Table 2. The meta-analysis comparing CS patients with HCs revealed decreased brain function in CS patients in the right superior temporal gyrus and left postcentral gyrus, and increased function in the left superior frontal gyrus (Figure 2). Egger’s test results for the right superior temporal gyrus (Bias 1.19, Z 1.00, P 0.318), left postcentral gyrus (Bias-0.02, Z-0.02, P 0.988), and left superior frontal gyrus (Bias 0.77, Z 0.73, P 0.467) confirmed no significant heterogeneity among the studies (Table 2). Jackknife sensitivity analysis showed consistent results, with the findings for the right superior temporal gyrus replicated in 13 of 16 iterations; for the left postcentral gyrus in 14 of 16; and for the left superior frontal gyrus in 14 of 16 iterations (Table 3). Meta-regression analysis indicated no significant impact from differences in age and gender between groups.

**Table 2:** Applying SDM method to study the changes in brain function activity cervical spondylosis.

	Anatomical label Brodmann area (BA)	Peak MNI coordinate			SDM-Z	*p* value	Voxels	Jacknife	Egger
		X Y Z						Sensitivity analysis	Bias test
ReHo/zReHo & ALFF/ /dALFF/zALFF Decrease									
	Right superior temporal gyrus BA 22	62	-38	8	−2.558	0.005259216	303	13/16	Bias: 1.19 Z: 1.00 P: 0.318
	Left postcentral gyrus BA4	−38	−26	54	−2.181	0.014583647	127	14/16	Bias: −0.02 Z: −0.02 P: 0.988
ReHo/zReHo & ALFF/ /dALFF/zALFF Increase									
	Left superior frontal gyrus BA 10	−6	66	0	2.447	0.007206142	1,451	14/16	Bias: 0.77 Z: 0.73 P: 0.467

ReHo, regional honogeneity; ALFF, amplitude of low-frequency fluctuation; fALFF, fractional amplitude of low-frequency fluctuation; MNI, montreal neurological institute.

**Table 3:** Results of sensitive analysis.

Discarded article	Decreased		Increased
	Right superior temporal gyrus	Left postcentral gyrus	Left superior frontal gyrus
Yue, X. 2013	N	Y	N
Zhou, F. 2014	Y	Y	Y
Tan, Y.M. 2015	Y	N	Y
Tan, Y. 2015	Y	N	Y
Yu, C.X. 2017	N	Y	Y
Chen, Z. 2018	Y	Y	Y
Chen, J. 2018	Y	Y	Y
Xu, Y.K. 2018	Y	Y	Y
Zhang, CL. 2019	Y	Y	Y
Ge, Z.C. 2021	Y	Y	Y
Wu, K.F. 2021	Y	Y	N
Fan, N.J. 2022	Y	Y	Y
Bai, L. 2022	Y	Y	Y
Zhao, R. 2022	Y	Y	Y
Su, Q. 2023	Y	Y	Y
Yue, X. 2013	N	Y	Y

Y, YES; N, NO.

## 4 Discussion

This study employs AES-SDM analysis to integrate brain functional activation maps, confirming notable changes in brain function in CS patients compared to HCs. Specifically, we observed decreased activation in the right superior temporal gyrus and the left postcentral gyrus, and increased activation in the left superior frontal gyrus.

### 4.1 Decreased brain function regions in CS patients

The right superior temporal gyrus, part of the Wernicke’s area involved in auditory and language processing and a key structure in social cognitive functions, showed decreased brain activity in this study. This region also acts as a cortical center for vestibular functions (28). Yu et al. (11) reported that the cognitive levels maintained in CS patients might be a result of compensatory mechanisms, which, upon failure, could lead to significant cognitive decline.

Furthermore, Song et al. (29) suggested that changes in gray matter volume in regions such as the left precentral gyrus, medial prefrontal cortex, supplementary motor area, superior temporal gyrus, parietal lobe, occipital lobe, and postcentral gyrus may indicate Wallerian degeneration, which occurs distal to damage in the corticospinal tract. Adaptive changes in sensory and motor functions have been confirmed in animal models with hemisected spinal cords (30). Wrigley et al. (31) also found significant reductions in gray matter volume in the left precentral gyrus, bilateral superior temporal gyri, insula, hypothalamus, medial prefrontal cortex, and anterior cingulate cortex in patients with spinal cord injury compared to healthy individuals. CS damages afferent or efferent fibers, such as those in the corticospinal tract, disrupting intact motor-related neural reflex-feedback control systems. Brain regions involved in motor activity, including the premotor and supplementary motor areas, play crucial roles in the preparation and execution of movement (32). Therefore, this study posits that the decreased brain function activity in the superior temporal gyrus in CS patients affects the intention, purpose, and rough planning of the motor area during movement execution, which may explain the fine motor abnormalities seen in CS patients.

The left postcentral gyrus, part of the primary sensory cortex, plays a crucial role in the pathological process of CS, integrating and executing various motor information and participating in the perception of touch, pressure, temperature, and pain (33–35). The observed decrease in brain function activity in the left postcentral gyrus suggests cortical reorganization in the cortical functional area (i.e., postcentral gyrus) following damage to the corticospinal tract nerve fibers, compensating for functional deficits in CS patients. Recent studies have also identified functional, structural, and metabolic changes in the postcentral gyrus of CS patients, considered manifestations of sensorimotor cortex plasticity, revealing dynamic adjustments by the brain in response to secondary damage during chronic spinal cord injury progression (36, 37). Moreover, previous literature indicates the postcentral gyrus’s association with chronic pain (38, 39). The regulation of external stimuli or neurofeedback can directly impact chronic pain, highlighting the significance of the sensorimotor cortex in the pain process. Our findings of decreased brain function activity in the left postcentral gyrus suggest that CS-related chronic neck and shoulder pain affects the functional expression of the somatosensory cortex, leading to weakened sensory abilities and long-term behavioral impacts. Thus, it can be inferred that the decrease in brain function activity may signify the activation of brain functions in the postcentral gyrus, thereby influencing pain perception.

### 4.2 Increased brain function regions in CS patients

The prefrontal cortex, encompassing areas such as the medial superior frontal gyrus and the orbitofrontal cortex, is responsible for a wide array of advanced cognitive functions and emotional regulation. It plays a crucial role in modulating the brain’s perception of and response to internal and external environmental changes, executive functions, cognitive control, language processing, and the retrieval of episodic memory. Additionally, it coordinates with other brain regions to maintain the fundamental activities of the brain in a resting state (40–42). A study by Wang et al. (43) found that, compared to the control group, patients with mild, moderate, and severe CS showed lower ALFF values in the left medial superior frontal gyrus. This suggests that the reduction in ALFF in the left medial superior frontal gyrus might be an underlying neuroimaging phenotype of CS, underscoring the significant role of the medial prefrontal cortex in regulating the dynamic interaction of emotional and cognitive signals in the brain (44). This revelation provides insights into the neurobiological basis of CS and highlights the importance of understanding changes in brain activity associated with CS. However, the impact of emotional states on the pre-motor behavioral responses related to ALFF changes warrants further investigation beyond the scope of the current study.

Additionally, research by Koenigs M et al. indicates that activity in the medial superior frontal gyrus is associated with the experience of negative emotions, playing a foundational role in the processing of negative emotions (45). In adolescent females with severe depression who have attempted suicide, an increase in ALFF in the right superior frontal gyrus has been observed (46, 47). Furthermore, changes in ALFF in regions including the right superior frontal gyrus are induced following electroconvulsive therapy in adolescents with depression, suggesting the right superior frontal gyrus as a potential neurobiological marker for clinical treatment (48). Therefore, a deeper understanding of the function of the medial superior frontal gyrus is crucial for exploring the pathogenesis and treatment approaches for diseases like CS. Future research will further elucidate the role of the medial prefrontal cortex in emotional and cognitive domains, offering more effective strategies for the diagnosis and treatment of neurological diseases.

Overall, this study demonstrates an increase in brain function activity in the left superior frontal gyrus in CS patients, providing further evidence of compensatory mechanisms distal to spinal structural damage in CS patients. It is reasonable to believe that the increased functional activity in the left superior frontal gyrus may play a significant role in neural plasticity. This might also explain a mechanism in some CS patients who, despite having clear evidence of neck compression and significant degenerative demyelination, are able to function normally with minimal or mild neurological deficits.

### 4.3 Limitation

This meta-analysis has several limitations that warrant cautious interpretation of the results. First, the number of studies included in the meta-analysis is relatively small, which may affect the generalizability and robustness of the findings. Secondly, our reliance on peak coordinates from published data may not comprehensively represent the spatial extent of brain activity alterations, introducing potential selection bias. Variability in imaging protocols and analysis methods across studies might influence the results, despite rigorous inclusion criteria and statistical approaches. Finally, some of the included studies involved patients who had received pharmacological treatment prior to MRI scanning.

## 5 Conclusion

This study, utilizing SDM-Meta analysis, identified decreased brain function in the right superior temporal gyrus and left postcentral gyrus, and increased function in the left superior frontal gyrus in patients with CS. These findings contribute to our understanding of the changes in brain regions associated with CS as reported in previous literature and provide a basis for further investigation into the central mechanisms of CS. Future research is needed to determine how these localized changes in neural activity can be applied to the diagnosis of the disease, monitoring disease progression, and developing potential therapeutic interventions for patients with CS.

## Data Availability

The original contributions presented in the study are included in the article/supplementary material, further inquiries can be directed to the corresponding author.

## Footnotes

^1^http://www.brainmap.org/icbm-2tal/

^2^www.sdmproject.com/utilities/?show=Statistics
